# Calcitonin gene-related peptide promotes cellular changes in trigeminal neurons and glia implicated in peripheral and central sensitization

**DOI:** 10.1186/1744-8069-7-94

**Published:** 2011-12-06

**Authors:** Ryan J Cady, Joseph R Glenn, Kael M Smith, Paul L Durham

**Affiliations:** 1 Center for Biomedical & Life Sciences, Missouri State University, 524 N. Boonville, Springfield, MO, USA

## Abstract

**Background:**

Calcitonin gene-related peptide (CGRP), a neuropeptide released from trigeminal nerves, is implicated in the underlying pathology of temporomandibular joint disorder (TMD). Elevated levels of CGRP in the joint capsule correlate with inflammation and pain. CGRP mediates neurogenic inflammation in peripheral tissues by increasing blood flow, recruiting immune cells, and activating sensory neurons. The goal of this study was to investigate the capability of CGRP to promote peripheral and central sensitization in a model of TMD.

**Results:**

Temporal changes in protein expression in trigeminal ganglia and spinal trigeminal nucleus were determined by immunohistochemistry following injection of CGRP in the temporomandibular joint (TMJ) capsule of male Sprague-Dawley rats. CGRP stimulated expression of the active forms of the MAP kinases p38 and ERK, and PKA in trigeminal ganglia at 2 and 24 hours. CGRP also caused a sustained increase in the expression of c-Fos neurons in the spinal trigeminal nucleus. In contrast, levels of P2X_3 _in spinal neurons were only significantly elevated at 2 hours in response to CGRP. In addition, CGRP stimulated expression of GFAP in astrocytes and OX-42 in microglia at 2 and 24 hours post injection.

**Conclusions:**

Our results demonstrate that an elevated level of CGRP in the joint, which is associated with TMD, stimulate neuronal and glial expression of proteins implicated in the development of peripheral and central sensitization. Based on our findings, we propose that inhibition of CGRP-mediated activation of trigeminal neurons and glial cells with selective non-peptide CGRP receptor antagonists would be beneficial in the treatment of TMD.

## Background

Peripheral and central sensitization are implicated in the pathology of temporomandibular joint disorder (TMD), which is a musculoskeletal condition characterized by pain and discomfort of the masticatory system including the temporomandibular joint (TMJ) and associated muscles [1,2]. TMD is a prevalent disorder with as much as 70% of the population having at least one TMD symptom and 3-7% of the population seeking treatment for the disorder [3,4]. Activation of trigeminal ganglia neurons, which provide sensory innervation to the joint and muscles of mastication, is implicated in TMD pathology by providing a nociceptive pathway [5]. In response to inflammatory or noxious stimuli, trigeminal ganglia neurons release neuropeptides and other molecules that initiate and maintain neurogenic inflammation in the peripheral tissue that facilitate peripheral sensitization of trigeminal nociceptors [6]. In addition, excitation of trigeminal ganglion neurons leads to activation of second order neurons and glia that promotes central sensitization, hyperalgesia, and allodynia [7]. Thus, the trigeminal system provides a nociceptive conduit between peripheral inflammation in the joint or muscles and activation of central pain pathways in TMD.

The 37 amino acid neuropeptide calcitonin gene-related peptide (CGRP), which is synthesized and released from trigeminal ganglia neurons, is proposed to play a central role in the underlying pathology of TMD [8,9]. CGRP-containing trigeminal nerve fibers are present in the synovial membrane, articular disk, periosteum, and joint capsule of the TMJ [10,11]. Importantly, elevated CGRP levels in TMJ synovial fluid are indicative of mobility impairment and pain associated with arthritis [12] and inflammation [13]. CGRP is thought to contribute to TMD pathology by promoting neurogenic inflammation within the capsule via its ability to regulate blood flow, recruit and activate immune cells [14], and sensitize and activate trigeminal nociceptors [15]. In this way, transient increases in CGRP levels would promote inflammation and pain within the joint, while chronically elevated levels would lead to destruction of the TMJ capsule. The pathophysiological effects of CGRP are likely to involve development of peripheral and central sensitization, which are characteristic of TMD pathology.

There is accumulating evidence that supports a central role of CGRP in the initiation and maintenance of peripheral and central sensitization [16-18] via stimulation of neuronal and glial activity within trigeminal ganglia and spinal trigeminal nucleus. The cellular effects of CGRP are mediated via activation of the CGRP receptor, which is expressed by neurons [19] and glia [20] in trigeminal ganglia, and second order neurons and astrocytes in the spinal cord and brainstem nuclei [19,21]. Importantly, the potent peptide CGRP receptor antagonist, CGRP_8-37 _has been shown to effectively inhibit vasodilation and neurogenic inflammation in animal models [22,23], and decrease pain thresholds for several days [24]. In addition, the role of CGRP in the development of nociceptive behaviors in response to peripheral inflammatory events has been confirmed in studies of CGRP knockout mice [25]. However, the cellular mechanisms by which CGRP promotes peripheral inflammation and nociception are not well understood. Thus, the goal of our study was to investigate changes in trigeminal ganglia and spinal trigeminal nucleus neurons and glia implicated in the development of peripheral and central sensitization in response to elevated levels of CGRP, as reported during TMJ pathology. Specifically, changes in the expression of the signaling molecules PKA, active ERK and p-38, and the purinergic ion channel P2X_3 _have all been reported to play important roles in joint inflammation and pain [26-29].

## Results

### CGRP increases neuronal expression of MAP Kinases P-p38 and P-ERK in Trigeminal Ganglia

**Figure 1:**
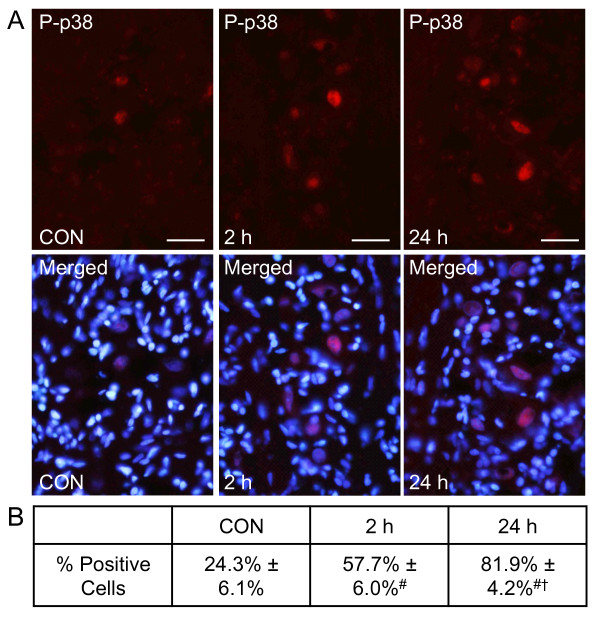
**Increased number of trigeminal ganglia neurons expressing P-p38 in response to CGRP injection into the TMJ capsule**. Sections of the posterolateral portion of the ganglion (V3) were obtained from untreated animals (CON), and animals receiving bilateral injections of CGRP after 2 and 24 hours. (A) Images of neuron-satellite glial regions stained for P-p38 are shown in the top panels. Magnification bar = 50 μm. The bottom panels are the same sections co-stained for P-p38 and DAPI. (B) The number of P-p38 positive cells ± SEM for each condition is reported (n = 3 independent experiments) ^#^*P *< 0.01 when compared to control levels, while ^† ^*P *< 0.01 when compared to CGRP stimulated levels at 2 hours.

Elevated levels of the phosphorylated active forms of the MAP kinases p38 and ERK are associated with development of peripheral sensitization [30]. To determine whether the expression of P-p38 and P-ERK in the trigeminal ganglia would be increased in response to bilateral TMJ injections of 1 μM CGRP, ganglia were isolated 2 hours and 24 hours post injection and P-p38 and P-ERK staining levels were compared to untreated animals. Changes in P-p38 expression were determined by performing cells counts of positively expressing neurons in the V3 region of the ganglia, which contains cell bodies of neurons that innervate the TMJ capsule. Data are expressed as a percentage of the total number of neurons as identified by the fluorescent nuclear dye DAPI. As seen in Figure 1, 24.30% ± 0.06 of neurons expressed P-p38 under basal unstimulated conditions. However, at the 2 hour time point, the number of neurons with positive P-p38 staining was significantly increased (57.69% ± 0.06, *P *< 0.01). Similarly the number of neurons staining positive for P-p38 at 24 hours was significantly increased (81.94% ± 0.04, *P *< 0.01) compared to both the control and 2 hour CGRP stimulation. These data provide evidence that CGRP activation of trigeminal neurons leads to a prolonged stimulatory effect on neuronal levels of P-p38.

**Figure 2:**
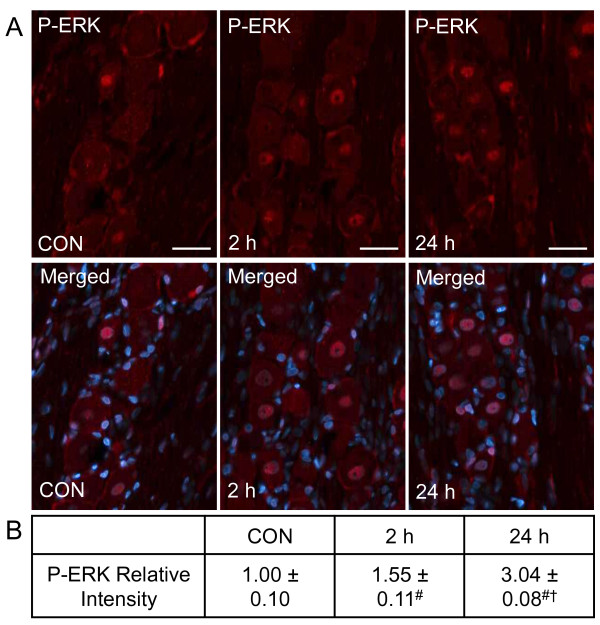
**CGRP stimulates increased P-ERK expression in trigeminal ganglia neurons and satellite glia**. Sections of the posterolateral portion of the ganglion (V3) were obtained from untreated animals (CON), or animals injected with CGRP in each TMJ capsule. (A) Images of neuron-satellite glial regions stained for P-ERK are shown in the top panels. Magnification bar = 50 μm. The bottom panels are the same sections co-stained for P-ERK and DAPI. (B) The average fold change ± SEM of P-ERK staining intensity from control values, whose mean was made equal to one, is reported (n = 3 independent experiments) ^# ^*P *< 0.01 when compared to control levels, while ^† ^*P *< 0.01 when compared to CGRP stimulated levels at 2 hours.

Similar to the P-p38 results, CGRP injection into the TMJ capsule increased expression of P-ERK in neurons. While staining was barely detectable in control ganglia, the expression of the P-ERK, as measured as a change in relative staining intensity, was increased primarily in neurons 2 hours after CGRP injection (Figure 2). However, CGRP was found to greatly stimulate P-ERK expression in both neurons and satellite glial cells within the V3 region of the ganglion at 24 hours. The increase in staining intensity (1.55 ± 0.11, *P *< 0.01) observed at the 2 hour time point post CGRP injection was significantly greater than control levels (1.00 ± 0.10), while CGRP levels at 24 hours were significantly higher (3.04 ± 0.11, *P *< 0.01) than the control and 2 hour values. Thus, elevated levels of CGRP in the joint capsule stimulate a small increase in the neuronal expression P-ERK at 2 hours but induce a much greater response in neurons and glia after 24 hours.

### CGRP increases neuronal expression of c-Fos in the spinal trigeminal nucleus

**Figure 3:**
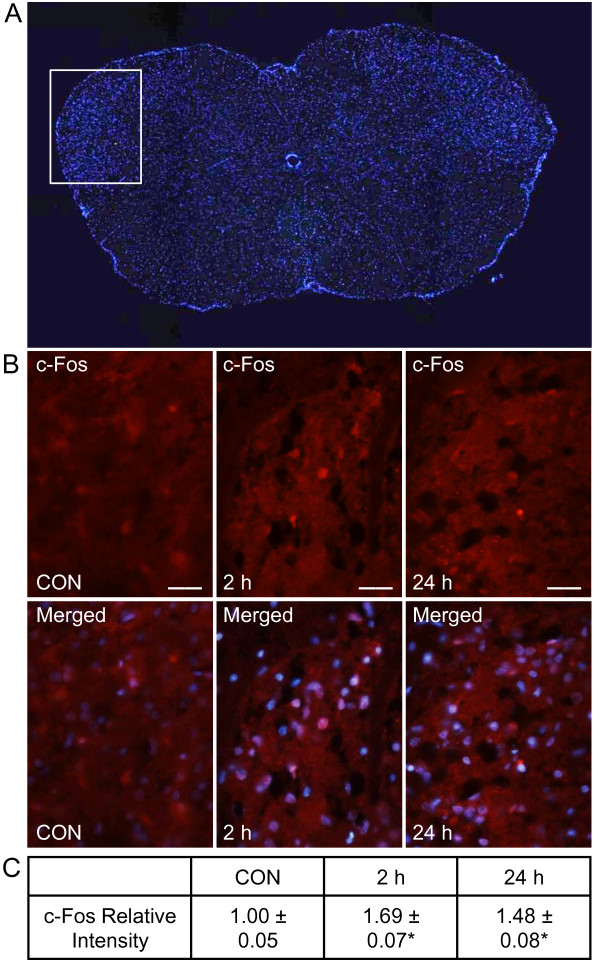
**Peripheral injection of CGRP induces a sustained increase in expression of neuronal c-Fos in spinal trigeminal nucleus**. (A) An image (40×) of a section of spinal cord 4 mm from the obex stained with the nuclear dye DAPI is shown. The white box represents the area encompassing the spinomedullary junction (Vc/C1-C2) transition zone. (B) Sections of spinal cord within the Vc/C1-2 region of the spinal trigeminal nucleus were obtained from control animals (CON), animals 2 hours post CGRP injections, or animals 24 hours post CGRP injection. Images of spinal cord tissues stained for c-Fos are shown in the top panels. Magnification bar = 50 μm. The same sections costained for c-Fos and DAPI are displayed in the bottom panels. (C) The average fold change ± SEM of c-Fos staining intensity from control values is reported (n = 3 independent experiments). * *P *< 0.05 when compared to control values.

Expression of c-Fos, a member of the early immediate family of transcription factors, was used to study the activation level of second order sensory neurons within the spinal medullary horn. Initially, tissues from the upper spinal cord containing the trigeminal nucleus caudalis (Vc/C1-2 region of the spinal cord 4-5 mm posterior to the obex) in unstimulated animals were stained with DAPI to identify the nuclei of neurons and glia (Figure 3A). Tissues containing the trigeminal nucleus caudalis were then stained with antibodies directed against c-Fos and costained with DAPI. As seen in Figure 3B-C, the number and relative intensity of neuronal

c-Fos immunoreactive cells was significantly greater at 2 hours (1.69 ± 0.07, *P *< 0.05) and 24 hours (1.48 ± 0.08, *P *< 0.05) post CGRP injection when compared to levels in untreated control animals (1.00 ± 0.05). Thus, elevated levels of CGRP in the TMJ capsule lead to activation of second order neurons within the spinal trigeminal nucleus.

### PKA and P2X_3 _expression in spinal trigeminal nucleus are elevated in response to CGRP

**Figure 4:**
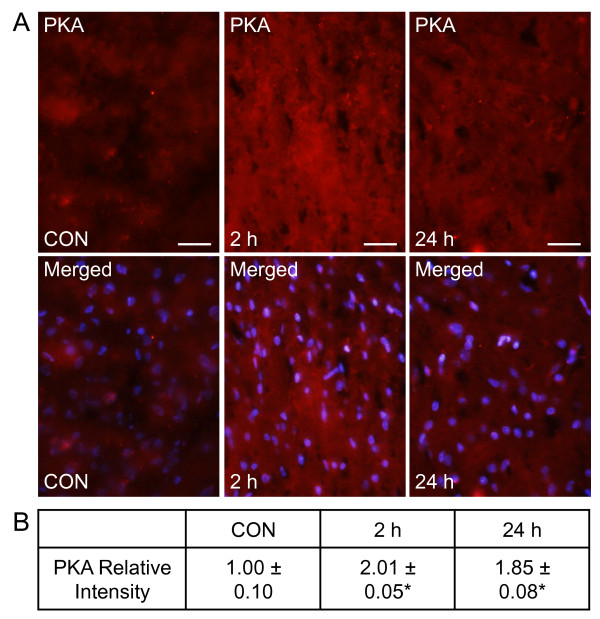
**PKA levels are elevated in the spinal trigeminal nucleus in response to CGRP**. Spinal cord sections were obtained from control animals (CON), animals 2 hours post CGRP injections, or animals 24 hours post CGRP injection. (A) Images of spinal cord tissues stained for PKA are shown in the top panels. Magnification bar = 50 μm. The same sections costained for PKA and DAPI are displayed in the bottom panels. (B) The average fold change ± SEM of PKA staining intensity from control values is reported (n = 3). * *P *< 0.05 when compared to control values.

The effect of peripheral CGRP on expression of the pro-inflammatory signaling protein PKA and purinergic receptor P2X_3_, whose expression correlates with nociceptive transmission, was investigated. As seen in Figure 4, low level expression of PKA (1.00 ± 0.10) was detected in tissues sections from the upper spinal cord containing the trigeminal nucleus caudalis. In contrast, the relative staining intensity for PKA was significantly increased in neurons and glia over control levels in tissues 2 hours (2.01 ± 0.05, *P *< 0.05) and 24 hours (1.85 ± 0.08, *P *< 0.05) post CGRP injection.

**Figure 5:**
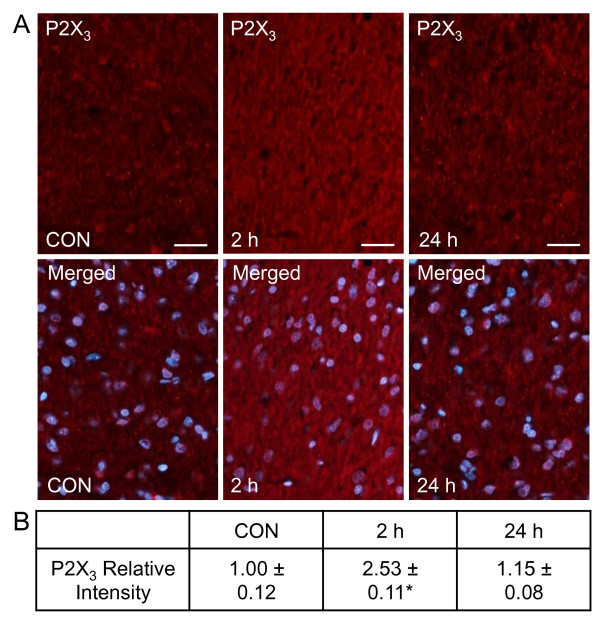
**CGRP causes a transient increase in P2X_3 _expression in the spinal trigeminal nucleus at 2 and 24 hours**. Sections of spinal cord were obtained from control animals (CON), animals 2 hours post CGRP injections or animals 24 hours post CGRP injection. (A) Images of spinal cord tissues stained for P2X_3 _are shown in the top panels. Magnification bar = 50 μm. The same sections costained for P2X_3 _and DAPI are displayed in the bottom panels. (B) The average fold change ± SEM of P2X_3 _staining intensity from control values is reported (n = 3). * *P *< 0.05 when compared to control values.

Similar to the findings with PKA, CGRP significantly stimulated neuronal P2X_3 _expression in the spinal trigeminal nucleus. While minimal P2X_3 _immunostaining was observed in control tissues (1.00 ± 0.12), the relative level of staining was greatly increased at 2 hours (2.53 ± 0.11, *P *< 0.05). However at 24 hours, P2X_3 _expression had returned to control levels (1.15 ± 0.08) (Figure 5). To confirm expression of P2X_3 _in neurons, some tissues were costained with antibodies directed against NeuN, which is a protein expressed in the nucleus of spinal cord neurons. Most of the NeuN positive neuronal cells in the outer lamina also expressed P2X_3 _(data not shown). Based on our findings, CGRP injection in the TMJ capsule leads to sustained increases in the levels of PKA in spinal trigeminal nucleus neurons and glia, and a transient elevation in P2X_3_.

### CGRP stimulates expression of OX-42 in microglia and GFAP in astrocytes

**Figure 6:**
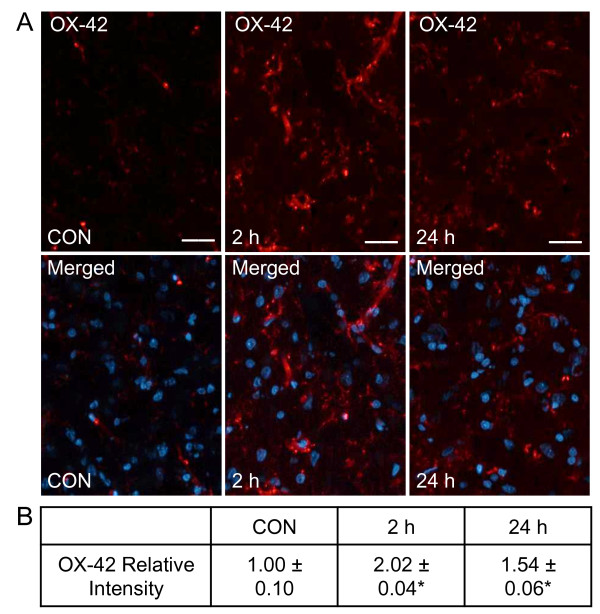
**Elevated expression of OX-42 in microglia**. Sections of spinal cord were obtained from control animals (CON), animals 2 hours post CGRP injections, or animals 24 hours post CGRP injection. (A) Images of spinal cord tissues stained for OX-42 are shown in the top panels. Magnification bar = 50 μm. The same sections costained for OX-42 and DAPI are shown in the bottom panels. (B) The average fold change ± SEM of OX-42 staining intensity from control values is reported (n = 3). * *P *< 0.05 when compared to control values.

The effect of CGRP on microglial activation was investigated using OX-42 antibodies. As seen in Figure 6, low level expression of OX-42 (1.00 ± 0.10) was detected in tissue sections from the upper spinal cord containing the trigeminal nucleus caudalis. In contrast, the relative staining intensity for OX-42 was significantly increased in tissues 2 hours (2.02 ± 0.04, *P *< 0.05) and 24 hours (1.54 ± 0.06, *P *< 0.05) post CGRP injection.

**Figure 7:**
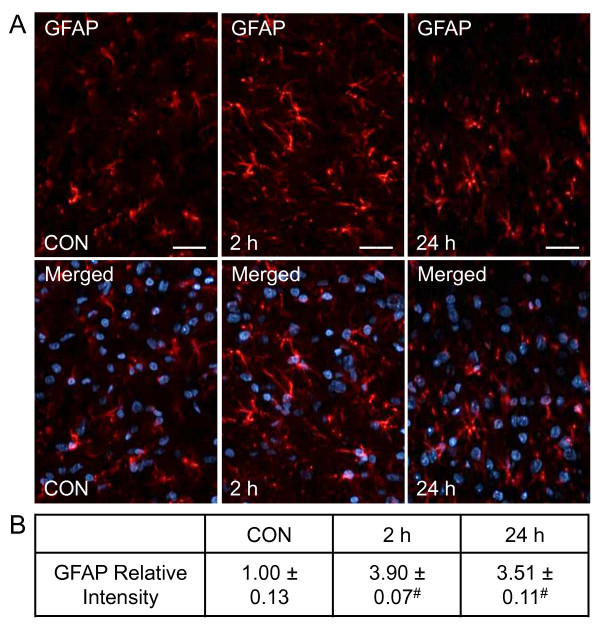
**CGRP induces a prolonged increase in expression of GFAP in astrocytes**. Spinal cord sections were obtained from control animals (CON), animals 2 hours post CGRP injections, or animals 24 hours post CGRP injection. (A) Images of spinal cord tissues stained for GFAP are shown in the top panels. Magnification bar = 50 μm. The same sections costained for GFAP and DAPI are displayed in the bottom panels. (B) The average fold change ± SEM of GFAP staining intensity from control values is reported (n = 3). ^# ^*P *< 0.01 when compared to control values.

Changes in expression of the cytoskeletal protein GFAP were used to determine the activity level of astrocytes in the medullary horn. As shown in Figure 7, control animals express a relatively low level of GFAP immunostaining (1.00 ± 0.13), while animals injected with CGRP exhibited a marked increase in GFAP immunoreactivity after 2 hours (3.90 ± 0.07, *P *< 0.001) that was maintained at 24 hours (3.51 ± 0.11, *P *< 0.001). Taken together, CGRP causes prolonged spinal trigeminal microglia and astrocyte activation.

## Discussion

In our study, we found that injection of CGRP into the TMJ capsule resulted in increased expression of proteins implicated in the development and maintenance of peripheral and central sensitization and nociception. The rationale for this study was based on reports that CGRP-containing trigeminal nerve fibers are present in the synovial membrane, articular disk, periosteum, and joint capsule of the TMJ [10,11] and high concentrations of CGRP in TMJ synovial fluid are indicative of mobility impairment and pain associated with arthritis [12] and inflammation [13]. The concentration of CGRP (1 μM) used in our study is similar to levels reported in TMJ exudates collected during inflammatory conditions [31,32]. Our finding that elevated levels of CGRP in the TMJ capsule can stimulate trigeminal neurons is in agreement with the proposed role of CGRP in TMD by promoting local inflammation as well as pain transmission from peripheral tissues to the CNS [33]. Towards this end, we found that CGRP stimulation of trigeminal neurons increased neuronal expression of P-p38 and P-ERK at 2 and 24 hours and increased P-ERK staining intensity in satellite glial cells in trigeminal ganglia at 24 hours. Both p-38 and ERK are members of the MAP kinase family of signal transduction enzymes activated in response to inflammatory stimuli, and are known to play an important role in the development of peripheral sensitization [28,34,35]. The MAP kinases are reported to mediate sensitization of primary and second order nociceptive neurons by increasing neuronal ion channel expression and activity, and expression of membrane receptors associated with nociception [28,36]. In addition, p38 and ERK are known to stimulate synthesis and secretion of cytokines from glial cells that promote and maintain a hyperexcitable state of neurons [6,28,37]. Further evidence of the importance of MAP kinases in the induction of peripheral sensitization and persistent pain was provided by results from studies in which blocking MAP kinase activity with specific inhibitors suppressed nociceptive responses and sensitization [28,38,39].

Results from our study provide evidence that elevated TMJ levels of CGRP can promote cellular events associated with the development of central sensitization. For example, we found that c-Fos expression in second order neurons within the spinal trigeminal nucleus was increased at 2 hours, and remained significantly elevated at 24 hours in response to bilateral injections of CGRP. In addition, upregulation of OX-42, a biomarker indicative of microglial activation [40], was observed at 2 and 24 hours after CGRP injection. CGRP also induced a large increase in expression of GFAP at 2 hours that remained at a similar elevated level at 24 hours. GFAP is an intermediate cytoskeleton filament protein selectively localized to mature astrocytes and, thus, serves as a biomarker of astrocyte activation [41]. Based on our findings, we propose that CGRP facilitates development of TMD by promoting an enhanced state of astrocyte and microglia activity, which is characteristic of central sensitization, persistent pain states, and nociceptive behaviors [6,42].

We also found that levels of PKA and P2X_3 _were elevated in response to CGRP injection into the TMJ capsule. However, while CGRP caused a more sustained increase in PKA expression in spinal neurons and glia, elevated levels of CGRP in the capsule resulted in a transient increase in neuronal expression of P2X_3. _Activation of intracellular signaling pathways involving PKA are known to play a key role in the induction and maintenance of central sensitization and persistent pain by phosphorylation of glutamate receptors and ion channels [43-45], and increasing expression of pro-inflammatory and pro-nociceptive genes. Furthermore, blocking PKA signaling results in reduction of inflammation-induced hyperalgesic behaviors [46,47]. Findings from our study provide evidence that neuronal levels of the purinergic receptor P2X_3 _were also significantly elevated at 2 hours after a single CGRP injection. Notably, the inflammatory and nociceptive effects of ATP are known to involve activation of P2X receptors, which are upregulated in sensitized nociceptive neurons [48-50]. In particular, activation of heteromeric P2X_2/3 _or homomeric P2X_3 _receptors, which are abundantly expressed by trigeminal ganglion neurons [51] is reported to mediate acute and chronic pain in response to inflammation or nerve injury [49,52-55]. Based on data from prior studies, we propose that elevated peripheral levels of CGRP increase membrane expression and sensitization of P2X_3 _receptors on second order neurons. Taken together, our findings demonstrate that elevated levels of CGRP, as reported in TMD, promote cellular changes in spinal trigeminal neurons and glia that temporally correlate with initiating and promoting central sensitization.

## Conclusions

In this study, we provide evidence that elevated levels of CGRP leads to cellular changes in proteins implicated in the development and maintenance of peripheral and central sensitization. Although not directly demonstrated in our study, we speculate that CGRP stimulation of MAP kinases, PKA, and P2X_3 _would also lead to increased nociceptive responses to thermal, mechanical, and chemical stimuli. Interestingly, data from recent phase II clinical studies provide evidence that a non-peptide CGRP receptor antagonist was effective as an abortive therapy for migraine [56], a disease that involves activation of trigeminal neurons, elevated levels of CGRP, and peripheral and central sensitization. Thus, based on our findings as well as others, we postulate that blocking the cellular effects of CGRP with the use of non-peptide antagonists would be beneficial in the treatment of TMD.

## Methods

### Animals

All animal studies were approved by the Institutional Animal Care and Use Committee at Missouri State University and were conducted in compliance with all established guidelines in the Animal Welfare Act and National Institutes of Health. Adult male Sprague-Dawley rats (200-250 grams; Charles River Laboratories, Wilmington, MA) were housed in structurally sound, clean plastic cages on a 12 hour light/dark cycle with unrestricted access to food and water. A concerted effort was made to reduce the suffering and number of animals used in this study. In addition, food and water consumption, weight, and grooming behaviors were recorded daily to monitor the overall health of the animals.

### CGRP injection as a model of TMJ inflammation

Rats were anesthetized by inhalation of 3.0% isoflurane (VetEquip, Pleasanton, CA). For the CGRP studies, rat CGRP (American Peptide, Sunnyvale, CA) was injected into each TMJ capsule (25 μl per injection; 1 μM in sterile water), while some animals were left untreated and served as controls. Rats were sacrificed by CO_2 _asphyxiation either 2 hours or 24 hours post injection.

### Tissue isolation and preparation

Trigeminal ganglia and spinal cord from the spinomedullary junction (Vc/C1-2) transition zone containing the trigeminal nucleus caudalis were removed from all rats following CO_2 _asphyxiation. Tissues were placed in a solution of 4% paraformaldehyde overnight followed by incubation in 15% sucrose in water at 4°C for 1 hour and then 30% sucrose overnight at 4°C. Trigeminal ganglia and spinal cord tissues were mounted with OCT Compound (Sakura Finetek, Torrance, CA) such that the ventral surface of the tissue was in contact with the slide, quickly frozen, and stored at -20°C. Fourteen-micron longitudinal sections of the entire trigeminal ganglion tissue were serially prepared using a cryostat (Microm HM 525, Thermo Scientific, Waltham, MA) set at -20°C. Spinal cord tissue containing spinal trigeminal nucleus was sectioned transversely at a distance of 4-5 mm posterior to the obex in 20 μm tissue sections using a cryostat set at -18°C. All sections were mounted on Superfrost Plus microscope slides (Fischer Scientific, Pittsburg, PA). Each slide used for immunohistochemistry contained at least one section from each experimental condition.

### Immunohistochemistry

Slides containing trigeminal ganglia or spinal cord tissue were permeabilized with 0.1% Triton X-100 plus 5% donkey serum in PBS for 20 minutes. Trigeminal ganglia sections were incubated overnight at 4°C with a P-p38 rabbit monoclonal antibody (1:200 in 5% donkey serum/PBS; Cell Signaling, Beverly, MA) or a P-ERK rabbit polyclonal antibody (1:200; Bioworld, St. Louis Park, MN). Spinal cord sections were incubated for 3 hours at room temperature with a mouse GFAP monoclonal antibody (1:500; Dako, Glostrup Denmark), mouse PKA polyclonal antibodies (1:100; BD Biosciences, San Jose, CA), rabbit c-Fos polyclonal antibodies (1:200; Abcam, Inc., Cambridge, MA), rabbit P2X_3 _polyclonal antibodies (1:1000; ThermoScientific, Rockford, IL), mouse NeuN monoclonal antibody (1:1000; Millipore), or a mouse OX-42 monoclonal antibody (1:200; Abcam). All sections were incubated for 1 hour at room temperature with either Alexa Fluor 594 donkey anti-mouse (PKA, GFAP, OX-42, NeuN) or rabbit (P-p38, P-ERK, c-Fos, P2X_3_; Invitrogen, Carlsbad, CA) diluted 1:500 in PBS, to detect immunofluorescent proteins by UV-fluorescence microscopy. Sections were costained with the nuclear dye 4'6-diamidino-2-phenylindole (DAPI; Vector Laboratories, Burlingame, CA,) and mounted in Vectashield (H 1200; Vector Laboratories). Images were collected from both trigeminal ganglia and both sides of spinal cord tissues at 400 × magnification using an Olympus DP70 camera mounted on an Olympus BX41 fluorescent microscope (Olympus, Center Valley, PA) or a Zeiss Axiocam mRm camera mounted on a Zeiss Imager Z1 fluorescent microscope equipped with an ApoTome.

### Measurement of cell counts and staining intensity

Images containing the mandibular branch (V3) of the middle portion of the trigeminal ganglion or regions of spinal cord tissue containing the trigeminal nucleus caudalis were used for analysis. Three images were taken from 3 independent experiments, resulting in 9 images for all cell count or intensity measurements, which were performed by two researchers blinded to the experimental conditions. To determine changes in expression of P-p38 in ganglia, the number of P-p38 positive neurons under each experimental condition were counted and expressed as a ratio of the total number of neurons identified by DAPI staining in each field. To quantify the staining intensity of P-ERK in the trigeminal ganglia, the mean gray intensity of 3 circular regions from areas containing a single neuron and associated surrounding satellite glial cells were measured and the mean gray intensity from an area containing only Schwann cells and fiber tracts was subtracted as background. To evaluate the expression of proteins in the trigeminal nucleus caudalis, images consisting of consecutive non-overlapping regions, containing cells from lamina I-IV were analyzed for each experimental condition. The staining intensity in spinal cord tissue was determined by measuring the mean gray intensity from 3 regions of staining in the trigeminal nucleus caudalis and subtracting the intensity from areas containing only background staining. The relative staining intensity measurements were determined using Image J software (Ver 1.43, Wayne Rasband, National Institutes of Health, Bethesda, MD) and based on our previously published protocols [26,57,58]. The fold change in staining intensity was defined as the mean change in relative intensity in the experimental condition when compared to mean levels of the unstimulated control tissue, which was set equal to one. Statistical analysis was performed using the non-parametric Mann-Whitney U test. Results were considered significant when *P *< 0.05. All statistical tests were performed using SPSS (Version 16, IBM, Chicago, IL).

## Competing interests

Paul Durham has served as a consultant and has received grant funding from Merck & Co. The other authors declare that they have no competing interests.

## Authors' contributions

RC contributed to data acquisition and analysis and preparation of the final manuscript; JG contributed to experimental design and acquisition of data; KS contributed to acquisition of data and manuscript preparation; PD contributed to experimental design, analysis and interpretation of data, and drafted as well as critically revised the manuscript. All the authors read and approved the final manuscript.
